# Close encounters of the fatal kind: Landscape features associated with central mountain caribou mortalities

**DOI:** 10.1002/ece3.7190

**Published:** 2021-02-04

**Authors:** Tracy L. McKay, Karine E. Pigeon, Terrence A. Larsen, Laura A. Finnegan

**Affiliations:** ^1^ fRI Research Caribou Program Hinton AB Canada; ^2^ University of Northern British Columbia Prince George BC Canada; ^3^ Yellowstone to Yukon Conservation Initiative Canmore AB Canada; ^4^ fRI Research Grizzly Bear Program Hinton AB Canada

**Keywords:** caribou, caribou habitat restoration, linear disturbance features, pipelines, predation risk, seismic lines

## Abstract

In western Canada, anthropogenic disturbances resulting from resource extraction activities are associated with habitat loss and altered predator–prey dynamics. These habitat changes are linked to increased predation risk and unsustainable mortality rates for caribou (*Rangifer tarandus caribou*). To inform effective habitat restoration, our goal was to examine whether specific linear disturbance features were associated with caribou predation in central mountain caribou ranges. We used predation‐caused caribou mortalities and caribou GPS‐collar data collected between 2008 and 2015 to assess caribou predation risk within and outside of protected areas at four spatio‐temporal scales: habitat use during the (a) 30 days, (b) 7 days, and (c) 24 hours prior to caribou being killed, and (d) characteristics at caribou kill site locations. Outside of protected areas, predation risk increased closer to pipelines, seismic lines, and streams. Within protected areas, predation risk increased closer to alpine habitat. Factors predicting predation risk differed among spatio‐temporal scales and linear feature types: predation risk increased closer to pipelines during the 30 and 7 days prior to caribou being killed and closer to seismic lines during the 30 days, 7 days, and 24 hours prior, but decreased closer to roads during the 30 days prior to being killed. By assessing habitat use prior to caribou being killed, we identified caribou predation risk factors that would not have been detected by analysis of kill site locations alone. These results provide further evidence that restoration of anthropogenic linear disturbance features should be an immediate priority for caribou recovery in central mountain caribou ranges.

## Graphical abstract

Anthropogenic disturbances associated with resource extraction activities are linked to increased predation risk and unsustainable mortality rates for woodland caribou, but few studies have directly assessed the relationships between specific anthropogenic disturbances and predation‐caused caribou mortalities. We assessed the influence of habitat, terrain, and anthropogenic linear disturbance features on caribou predation risk, including data from caribou mortality sites and caribou habitat use prior to being killed by predators. We found that predation risk increased closer to pipelines, seismic lines, and streams. Within protected areas, predation risk increased closer to alpine habitat. Factors predicting predation risk differed among spatio‐temporal scales and between linear feature types.

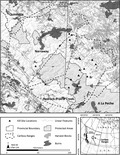

## 1 INTRODUCTION

Anthropogenic disturbance affects ecosystems across the globe (Haddad et al., 2015), and an essential consideration for wildlife management is understanding whether anthropogenic disturbances impact the survival of individuals, and ultimately, populations (Gill et al., 2001). In forested regions of western Canada, most anthropogenic landscape disturbances are associated with resource extraction activities, including forest harvest blocks, pipelines, roads, seismic lines, and wellsites. These features are linked to habitat loss, fragmentation, and changes in species interactions, including altered predator–prey dynamics (Bender et al., 1998; Schmiegelow & Monkkonen, 2002; Wittmer et al., 2007). In natural systems, predator–prey interactions are linked to spatial heterogeneity, including landscape characteristics that play a role in predator success (i.e., areas where prey are easier to catch) and landscape features that decrease predation risk (i.e., prey refugia or areas with higher escape probability) (Heithaus et al., 2009; Hopcraft et al., 2005; Kauffman et al., 2007). However, when anthropogenic disturbances affect prey vulnerability or change the habitat use or abundance of predators and prey, predator–prey dynamics can be altered to favor or hinder predator success, with potential population‐level effects for prey or predator species (DeMars & Boutin, 2018; Tucker et al., 2018).

Anthropogenic disturbance can enhance predation risk by increasing the spatial and temporal overlap between predators and prey (Frey et al., 2017; Whittington et al., 2011), increasing predator travel speeds and hunting efficiency (Dickie et al., 2017; Finnegan, Pigeon, et al., 2018; McKenzie et al., 2012), or decreasing the effectiveness of prey refugia (DeMars & Boutin, 2018; Peters et al., 2013). Anthropogenic disturbance can also increase predation risk through apparent competition, where habitat change alters the abundance and distribution of primary prey, resulting in an increased abundance and distribution of predators and increased predation rates for secondary prey species (DeCesare et al., 2010; Latham et al., 2011; Robinson et al., 2002).

Unnaturally high predation rates resulting from habitat alteration are considered the most significant and immediate threat to central mountain caribou (*Rangifer tarandus caribou*), which are designated as endangered by the Committee on the Status of Endangered Wildlife in Canada (COSEWIC, 2014, Environment Canada, 2014, Environment and Climate Change Canada, 2018). In addition to the management of critical caribou habitat, predator control and restoration of anthropogenic disturbance within caribou ranges are current priorities for caribou recovery efforts (Environment Canada, 2012, 2014; Government of Alberta, 2017a). However, despite the established link between anthropogenic disturbance and caribou predation risk (Festa‐Bianchet et al., 2011; Mumma et al., 2018; Whittington et al., 2011), relatively few studies have directly assessed the relationships between specific linear disturbance features (e.g., pipelines, roads, and seismic lines) and predation‐caused caribou mortalities (but see Apps et al., 2013; Latham et al., 2011; Mumma et al., 2017).

Because habitat restoration for caribou currently focuses on anthropogenic disturbance features (Environment Canada, 2012, 2014; Government of Alberta, 2017a), our objective was to determine whether specific anthropogenic linear disturbance features were directly associated with increased caribou predation risk. Using locations of predation‐caused caribou mortalities, live caribou GPS locations, and GIS‐based landscape variables, we assessed spatial variation in caribou predation risk in two central mountain populations in east‐central British Columbia and west‐central Alberta between 2008 and 2015, within and outside of parks and protected areas.

Studies evaluating caribou predation risk often focus on landscape characteristics at the kill site (e.g., Apps et al., 2013; James & Stuart‐Smith, 2000); however, caribou habitat use prior to being killed can influence their exposure to predation (Leblond et al., 2013; Peters et al., 2013) and alter their exposure to different predator species (Leblond et al., 2016). In our assessment of caribou predation risk, we investigated landscape attributes associated with habitat use prior to caribou being killed and attributes directly at kill site locations. In addition to anthropogenic disturbances, a range of other attributes could influence predation risk, including habitat characteristics (e.g., landcover) and terrain features (e.g., slope); therefore, we also included a number of habitat and terrain variables in our assessment. In accordance with previous research, we predicted that (a) caribou predation risk outside of protected areas would increase closer to all anthropogenic linear disturbance features (Apps et al., 2013; Latham, Latham, Boyce, et al., 2011; Mumma et al., 2017), and (b) caribou predation risk within protected areas would be linked to natural landscape characteristics associated with predator habitat selection (e.g., streams, forest edges, and lower elevations; DeCesare, 2012a; Knopff et al., 2014; Nielsen et al., 2006). We also predicted that variables influencing caribou predation risk at larger spatio‐temporal scales (i.e., habitat use prior to caribou being killed) would differ from those predicting predation risk at caribou kill site locations. Results from this study could be applied to focus caribou habitat restoration activities in areas with the highest predation‐caused caribou mortality risk, targeting the proximate cause of caribou declines (predation) in the rapidly declining caribou populations of western Canada (Hervieux et al., 2013; Johnson et al., 2015).

## 2 MATERIALS AND METHODS

### 2.1 Study area

**FIGURE 1:**
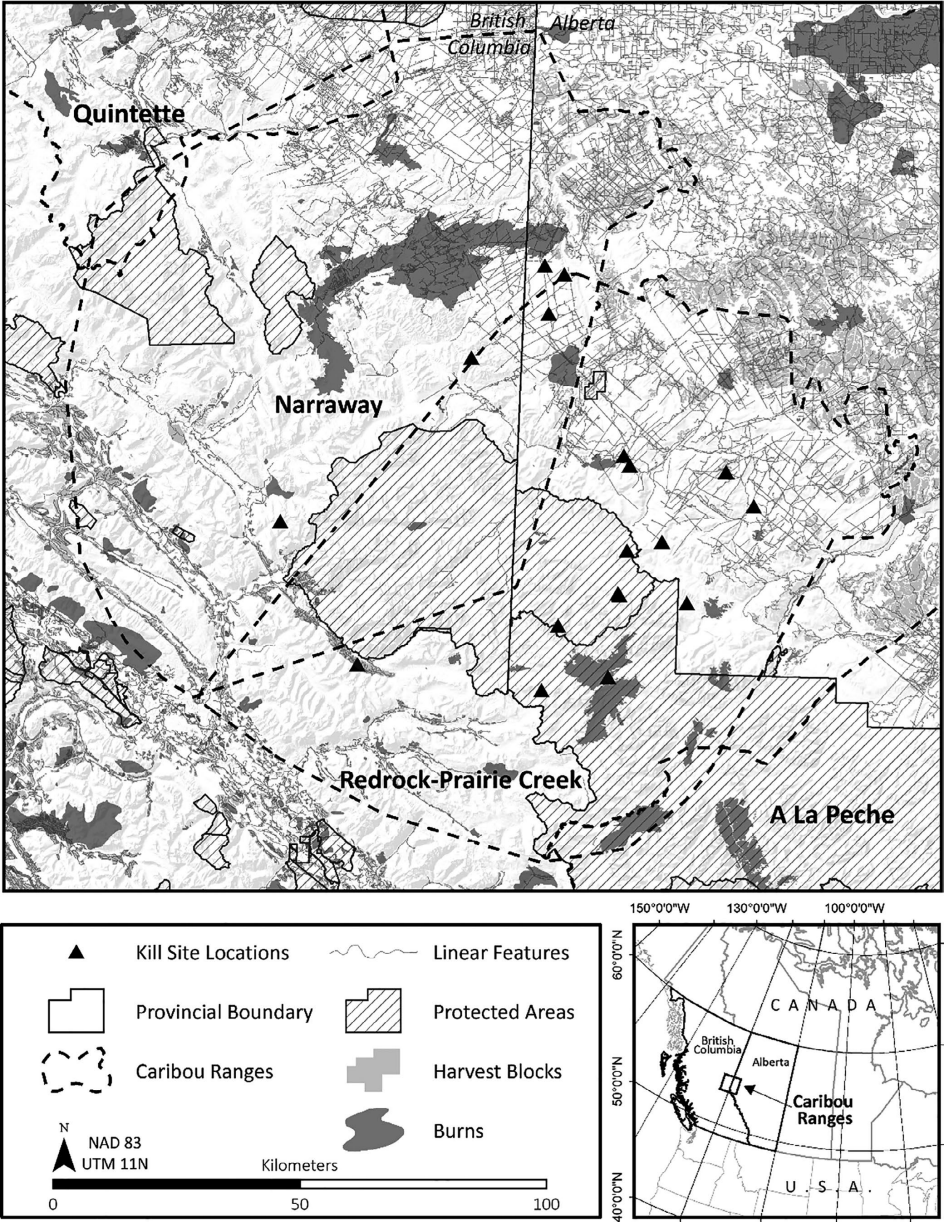
Study area in east‐central British Columbia and west‐central Alberta indicating predation‐caused caribou mortality locations (kill sites) recorded between 2009 and 2013 within the Narraway caribou range, and between 2008 and 2015 within the Redrock Prairie Creek caribou range. Two kill sites were only 450 m apart and are indicated with a 2. Anthropogenic linear disturbance features, forest harvest blocks, wildfires, and protected area boundaries are also shown, based on landscape conditions in 2015. Mean linear disturbance feature densities (5 km radius) are as follows: pipelines: within protected areas, 0 km/km^2^, outside of protected areas, 0.233 km/km^2^; roads: within protected areas, 0.002 km/km^2^, outside of protected areas, 0.457 km/km^2^; seismic lines: within protected areas, 0.003 km/km^2^, outside of protected areas, 0.460 km/km^2^

Our study area included the ranges of the Narraway and Redrock Prairie Creek central mountain caribou populations in east‐central British Columbia and west‐central Alberta (Figure 1; COSEWIC, 2011). Narraway and Redrock Prairie Creek caribou migrate between high elevation summer range (alpine and subalpine habitats) and low elevation winter range in the foothills (Brown & Hobson, 1998; COSEWIC, 2014; Edmonds, 1988). Alpine areas consist of exposed ridges and meadows with graminoid, sedge (*Carex spp*.), and herbaceous ground cover, and subalpine areas are characterized by Engelmann spruce (*Picea engelmannii*) and subalpine fir (*Abies lasiocarpa*), with dwarf shrubs (*Salix* and *Betula spp*.) along riparian zones. The foothills region consists of uplands dominated by lodgepole pine (*Pinus contorta*) and white spruce (*P. glauca*) and lowlands dominated by black spruce (*P. mariana*) and larch (*Larix laricina*; Natural Regions Committee, 2006, Demarchi, 2011). The study area includes provincial and national parks and protected areas (hereafter, “protected areas”) and public lands licensed for forestry and oil and gas development (Figure 1). Anthropogenic disturbances (forest harvest blocks, below‐ground pipelines, roads, seismic lines, and wellsites) are concentrated in the public lands in the eastern portion of the study area, and linear disturbance feature densities differ greatly within and outside of protected areas (Figure 1). Large scale natural disturbances in the region consist of wildfires. The predator–prey community includes gray wolves (*Canis lupus*), grizzly bears (*Ursus arctos*), black bears (*U. americanus*), cougars (*Puma concolor*), coyotes (*Canis latrans*), lynx (*Lynx canadensis*), wolverines (*Gulo gulo*), moose (*Alces alces*), white‐tailed deer (*Odocoileus virginianus*), mule deer (*O. hemionus*), elk (*Cervus elaphus*), mountain goats (*Oreamnos americanus*), bighorn sheep (*Ovis canadensis*), and caribou. Within the study area, eligible First Nations and Métis peoples hold hunting rights, and seasonal hunting licenses are also issued for wolves, black bears, cougars, coyotes, moose, white‐tailed deer, mule deer, elk, and sheep (Government of Alberta, 2019; Government of British Columbia, 2018). Grizzly bear hunting licenses were issued in British Columbia between 2008 and 2015, and trapping allocations were issued for wolves, coyotes, lynx, and wolverines (Government of Alberta, 2017b; Government of British Columbia, 2018). In the adjacent A La Peche caribou range in Alberta (Figure 1), a wolf population reduction program was initiated by the Alberta government in 2005 and continued throughout our study period (Government of Alberta, 2017a). In March 2014, delivery of limited wolf control actions occurred in the Redrock Prairie Creek caribou range on a trial basis, and full program delivery began during the 2014/2015 winter season (D. Hervieux, Alberta Environment and Parks, pers. comm.). Wolf control activities in the adjacent Quintette caribou range in British Columbia were initiated in the winter of 2015 (Seip & Jones, 2016).

### 2.2 Caribou locations

We used mortality and GPS location data collected from adult female caribou during 2009 to 2013 in the Narraway population (*n* = 24) and 2008 to 2015 in the Redrock Prairie Creek population (*n* = 34). Collar models were Televilt Iridium and Lotek 2200, 3300, and 4400 (Televilt Global Positioning System, Lindesberg, Sweden; Lotek Wireless Inc., Newmarket, Ontario, Canada). Caribou were captured and collared by the Government of Alberta via helicopter net‐gunning; capture and handling protocols were approved under Alberta Animal Care Protocol 008.

Caribou collars were programmed to send a mortality signal after eight consecutive hours of caribou immobility. Between 2008 and 2010, mortality events were detected during routine telemetry flights, and after 2010, mortality alerts were received immediately via satellite messages. Field crews investigated 37 mortality events in the field to determine probable cause of death (e.g., predation, accident, disease, and senescence) and to confirm accurate mortality locations. At each mortality site, field crews collected standardized data including the position, condition and distribution of the carcass, wound patterns, and identification of predator scat, prints, and sign (e.g., digging and bedding). As our objective was to investigate factors specifically related to predation‐caused caribou mortalities, for our analysis we only considered mortalities where field crews recorded strong field evidence of predation (*n* = 26). Because we required fine‐scale spatio‐temporal data for analysis, we also excluded mortality events if we could not accurately determine the date and time of death to within a 4‐hr period. We included only three‐dimensional (3D) locations in our analysis as average error distances for two‐dimensional fixes are generally higher than for 3D fixes (Frair et al., 2010; Sager‐Fradkin et al., 2010). Our final mortality dataset included 18 mortality events with predation as the probable cause of death and that were accurate relative to time of death and location. Although there was definitive evidence of specific predator species at a number of caribou mortality sites (e.g., wound patterns characteristic of a specific predator), we were not 100% confident in determining the predator species responsible for the initial kill in a number of the caribou mortalities investigated in the field, mainly due to delays in visiting the mortality site. Based on this uncertainty, relatively low sample sizes, and our interest in describing general caribou predation risk for management purposes, we did not assess predation risk from specific predator species.

The goal of our analysis was to inform caribou habitat restoration and management actions. Anthropogenic disturbances are almost entirely absent from protected areas in central mountain caribou ranges (Figure 1), and regions outside of protected areas are a likely area of focus for future management actions. Based on the potential for habitat restoration in regions outside of protected areas, and to compare factors influencing predation risk in areas with and without anthropogenic disturbance, we further partitioned the final caribou mortality dataset into locations within and outside of protected areas.

### 2.3 Landscape variables

We assessed a number of landscape variables in our analysis representing habitat, terrain, and anthropogenic linear disturbance features (hereafter, “linear disturbance features,” Table 1). We selected variables based on previous studies of caribou mortalities (e.g., Apps et al., 2013; James & Stuart‐Smith, 2000; Latham, Latham, Boyce, et al., 2011), predictors of predator occurrence (e.g., DeCesare, 2012a; Knopff, 2011; Nielsen et al., 2006), and specific linear disturbance features with the potential for restoration in central mountain caribou ranges (Environment Canada, 2012, 2014; Government of Alberta, 2017a). Because caribou and predator responses to landscape characteristics are known to be scale‐dependent (Ciarniello et al., 2007; DeCesare et al., 2012; Husseman et al., 2003), we investigated distance‐to‐feature variables at five spatial scales for most landscape variables. Distance‐to‐feature variables included Euclidian distance to features (m) and four exponential decay distances (1‐exp^(−n x distance (m)^) to represent the diminishing effect of features on animal response at greater distances, where the effect of distance becomes approximately constant at 1 km, 2 km, 5 km, and 10 km (Table 1).

**TABLE 1:** Model categories and variables considered in our assessment of predation risk for caribou in the Narraway and Redrock Prairie Creek ranges in east‐central British Columbia and west‐central Alberta between 2008 and 2015

Model category	Variables	Description	Range
Habitat	Alpine habitat	Distance to alpine habitat in m	0–40,585
		Exponential decay distances approaching constant (1.00) at 1, 2, 5, and 10 km	0–1
	Canopy cover	Canopy cover, %	0–100
	Forest	Presence/absence of forest cover (binary)	0 or 1
	Forest edge	Distance to nearest forest edge in m	0–1,642
		Exponential decay distances approaching constant (1.00) at 1, 2, 5, and 10 km	0–1
	Land cover	Dominant land cover/vegetation type (categorical)	1. Conifer (reference category);
			2. Deciduous/mixed forest/shrub/herbaceous;
			3. Alpine vegetation; 4. rock and ice
	Streams	Distance to nearest stream in m, Strahler order ≥3	0–4,389
		Distance to nearest stream in m, all streams	0–2,213
		Exponential decay distances approaching constant (1.00) at 1, 2, 5, and 10 km	0–1
Terrain	Aspect	South‐facing slopes versus all other aspects	0 or 1
	CTI	Compound topographic index (unitless)	1.44–24.6
	Elevation	Digital Elevation Model (DEM) (m)	570–3,259
	Slope	Slope angle (^o^)	0–82.7
	TPI	Topographic position index (unitless)	−6.68 to 12.32
Anthropogenic linear disturbance features	Pipelines	Distance to nearest pipeline in m	0–88,354
		Exponential decay distances approaching constant (1.00) at 1, 2, 5, and 10 km	0–1
	Roads	Distance to nearest road in m	0–52,372
		Exponential decay distances approaching constant (1.00) at 1, 2, 5, and 10 km	0–1
		Interaction between distance to road (m) and road density (5 km radius, 30 day data only)	0–3,079
	Seismic lines	Distance to nearest seismic line in m	0–26,062
		Exponential decay distances approaching constant (1.00) at 1, 2, 5, and 10 km	0–1
	Pipelines + Seismic	Distance to nearest pipeline or seismic line in m	0–88,354
		Exponential decay distances approaching constant (1.00) at 1, 2, 5, and 10 km	0–1

Decay distances were calculated to represent the diminishing effect of features on animal response at greater distances.

For habitat variables, we used landcover derived from airborne laser scanning and LandSat imagery (Nijland et al., 2015) and from LandSat imagery captured in 2000 (Canadian Forest Service Earth Observation for Sustainable Development of Forest (EOSD) cover map; Natural Resources Canada, 2009). We reclassified landcover into a categorical variable with four classes: (a) conifer, (b) deciduous/mixed forest/shrub/herbaceous, (c) alpine vegetation, and (d) nonhabitat (i.e., rock and ice) (Table 1). We also used landcover to create a binary variable representing the presence/absence of forest, to determine the distance to forest edges, and to calculate the distance to alpine habitat at the five spatial scales described above. We obtained streams data from the British Columbia Freshwater Atlas (GeoBC, 2016) and the Alberta Single Line Hydrography Network (GeoDiscover Alberta, 2019), and calculated distances to large streams and rivers (3rd Strahler order and higher), and distance to all streams in the study area, again at five spatial scales (Table 1). We used canopy cover (percent cover) derived from Landsat 7 imagery (McDermid, 2005).

To investigate the influence of terrain features, we used a 30 m × 30 m resolution digital elevation model (DEM) to extract values of elevation, aspect, slope, terrain wetness (compound topographic index, CTI; Gessler et al., 2000), and topographic position index (TPI); positive TPI values indicate ridges or hilltops, whereas negative values represent valley bottoms (Jenness, 2006). We calculated aspect as a binary variable representing south‐facing slopes (1) versus all other aspects (0).

We obtained open source data provided by the governments of Alberta (https://maps.alberta.ca/genesis/rest/services/Access/Latest/MapServer) and British Columbia (https://catalogue.data.gov.bc.ca) for linear disturbance features. We were interested in the separate influences of pipelines, roads, and seismic lines on predation risk for caribou; however, pipelines are often directly adjacent to roads. To isolate the influence of pipelines on caribou predation risk, we generated a pipeline dataset that excluded pipelines within 30 m of roads. Due to changes in the disturbance footprint within the study area between 2008 and 2015, we generated annual datasets for pipelines and roads. We included only conventional seismic lines (>5 m in width; hereafter “seismic lines”) in our analysis. Seismic lines were constructed prior to 2006; therefore, we generated a single dataset for seismic lines that was applied across all years of analysis. We calculated the distances to all linear disturbance features at the five spatial scales previously described. We also generated a combined linear disturbance feature variable that included pipelines and seismic lines grouped together, and based on preliminary results, we investigated an interaction (functional response) between distance to road (m) and road density (5 km radius) for 30‐day data outside of protected areas. Variables are described in Table 1.

### 2.4 Model building and analysis

In our investigation of predation risk, we were interested in assessing both caribou habitat use prior to predation events and landscape characteristics at caribou kill sites. Therefore, similar to Leblond et al., (2013), we considered a number of spatio‐temporal scales in our analysis. To investigate longer‐term associations between caribou habitat use and predation risk, we used GPS locations collected 30 days prior to caribou being killed as our largest spatio‐temporal scale. Average time between kills for wolves and cougars is approximately 7 days (Anderson & Lindzay, 2003; Merrill et al., 2010), and wolves circulate through territories approximately once every 7 days (Jędrzejewski et al., 2001). Therefore, we used GPS locations collected 7 days prior to caribou being killed for our intermediate spatio‐temporal scale. Prey detection distance for wolves has been reported at 1.0–1.5 km (Muhly et al., 2010; Whittington et al., 2011), similar to the average distance traveled in 24 hr by caribou in our study area (0.82–2.72 km, MacNearney et al., 2016). Consequently, we used GPS locations collected 24 hr prior to caribou being killed as our finest spatio‐temporal scale representing habitat use prior to predation events. For all three scales, we matched live GPS locations from nonsurviving caribou with GPS locations from all surviving caribou within the same herd range, matching live and nonsurviving locations during the same specific 30‐day, 7‐day, and 24‐hr period prior to the date and time of each caribou being killed. Finally, we investigated caribou kill sites, comparing the kill site location of nonsurviving caribou to locations from caribou within the same herd range that were alive during the specific date and time each caribou was killed. To account for variable collar fix rates (i.e., between 1 to 4 hr between fixes), we randomly selected one location from each surviving caribou collected up to 4 hr prior to the time of the nonsurviving caribou being killed. We used the ‘raster’ package in R (Hijmans, 2014) to extract annual landscape variables to caribou locations.

We carried out all data exploration and analyses within R (R Development Core Team, 2019) and RStudio (RStudio Team, 2020). Prior to analysis, we standardized variables by centring and scaling, using the ‘stdize’ function (MuMIn package, Bartón, 2015). We used a case–control approach, using conditional logistic regression models (survival package, Therneau, 2015) to assess predation risk, including the four spatio‐temporal scales (30 days, 7 days, and 24 hr prior to the caribou being killed, and at kill site locations), pairing nonsurviving caribou (case) with caribou that survived (control) during the same specific time period. We used the quasi‐likelihood under the independence model criterion (QIC) (‘MuMin’ package, Bartón, 2015) for model selection (Craiu et al., 2008; Pan, 2001).

For model selection, we grouped variables into three biologically relevant model categories: habitat, terrain, and linear disturbance features (Table 1). Our initial dataset included a large number of predictor variables and spatial scales with the potential to influence caribou predation risk (Table 1). In order to avoid a large number of *a priori* models and potential issues caused by uninformative parameters (Arnold, 2010), we carried out model selection using a four‐step process. First, for each spatio‐temporal scale, we built univariate models and selected the most informative spatial scale of effect for each feature (i.e., distance‐to‐feature in m, exponential decay distances at 1 km, 2 km, 5 km, and 10 km) based on QIC values. Second, we tested continuous variables (carried forward from step 1) for correlation using Pearson's R, and reviewed boxplots and variance inflation factors (VIFs) for categorical and binary variables. If variables were correlated (correlation coefficient ≥ 0.5 or VIF > 3), we compared variables in a univariate regression analysis and used QIC values to select the most informative variables to carry forward into step 3. Third, for each model category (habitat, terrain, and linear disturbance features), we compared a set of models including all possible combinations of variables (carried forward from step 2), and selected the most parsimonious model (i.e., lowest QIC values) for each model category. Candidate variables and final models for each model category at each spatio‐temporal scale outside of and within protected areas are listed in Appendix 1. Last, we compared all possible combinations of the final habitat, terrain, and linear disturbance feature models (selected in step 3) using QIC values and model weights (*ω*_i_) to identify the most parsimonious model(s) for each spatio‐temporal scale (i.e., 30 days, 7 days, and 24 hr prior to the caribou being killed, and at kill site locations), both within and outside of protected areas. When combining habitat, terrain, and linear disturbance feature models, we compared any correlated variables between model categories in a univariate regression analysis, and used QIC values to select the most informative variables to retain in combined models. If the weight (*ω*_i_) of the top model was <0.9, we carried out model averaging of all models with delta QIC values < 2 (Grueber et al., 2011). We report all results as standardized beta coefficients (*β*) with 95% confidence intervals as calculated from models selected in step 4 of the model building process.

We assessed model accuracy using the C‐statistic, the estimated conditional probability that for any pair of “case” and “control,” the predicted risk of an event is higher for the case than for the control; the C‐statistic is equivalent to the receiver operating characteristic (ROC) area under the curve (AUC) (Austin & Steyerberg, 2012; Steyerberg et al., 2010; Uno et al., 2011). Values of 0.9 and above represent high model accuracy, between 0.7 and 0.9 indicate good accuracy, and values < 0.7 indicate low model accuracy (Manel et al., 2001; Swets, 1988). We also assessed the predictive ability of each final model with k‐fold cross‐validation using the hab package (Basille, 2015). We randomly partitioned our strata into 80% training data and 20% testing data, and calculated the correlation (*r*_s_) between the relative probabilities of observed and predicted data for GPS locations from nonsurviving caribou and surviving caribou. We ran 100 iterations, and we report the average and 95% confidence intervals of *r*_s_ values across all 100 comparisons, with better model performance indicated by higher values of *r*_s_.

## 3 RESULTS

We determined accurate mortality dates and times for 18 predation‐caused caribou mortality events between 2008 and 2015, including 12 mortalities outside of protected areas and 6 within protected areas. From data collected at mortality site investigations, we determined that cougars, wolves, and grizzly bears were the predators most likely responsible for 3, 4, and 6 predation‐caused caribou mortalities, respectively, with tracks and sign from more than one predator observed at 5 mortality sites. The dataset available for analysis included 36,139 GPS locations from 58 surviving caribou (27,601 outside of protected areas and 8,538 locations within protected areas) and 8,277 premortality GPS locations from 18 nonsurviving caribou (6,680 outside of protected areas and 1,597 locations within protected areas). Data exploration indicated highly skewed distributions and near‐zero densities and variances of linear disturbance features within protected areas (pipelines: mean density, 5 km radius = 0 km/km^2^, variance = 0, roads: mean density = 0.002 km/km^2^, variance = 0.00009, seismic lines: mean density = 0.003 km/km^2^, variance = 0.00016); therefore, we did not include linear disturbance features in models for protected areas. The most parsimonious models for each model category at each spatio‐temporal scale outside of and within protected areas are reported in Appendix 1. Final models at some spatio‐temporal scales included uninformative variables, and only coefficients with 95% confidence intervals that do not overlap zero are described below.

Coefficients for models outside of protected areas are in Table 2. Outside of protected areas, the most parsimonious model (*ω*_i_ = 1.00) predicting predation risk during the 30 days prior to caribou being killed indicated that nonsurviving caribou were more likely to be closer to pipelines and seismic lines, but further from roads when compared to surviving caribou. Nonsurviving caribou were also more likely to be closer to streams, and less likely to be on south‐facing slopes. The most parsimonious model (*ω*_i_ = 0.996) predicting predation risk during the 7 days prior to caribou being killed also indicated that nonsurviving caribou were more likely to be closer to pipelines, seismic lines, and streams, and less likely to be on south‐facing slopes when compared to surviving caribou. The most parsimonious model (*ω*_i_ = 1.00) predicting predation risk during the 24 hr prior to caribou being killed indicated that nonsurviving caribou were more likely to be closer to seismic lines, streams, and forest edges, and less likely to be on south‐facing slopes when compared to surviving caribou. The most parsimonious models predicting predation risk at kill site locations included TPI and distance to roads and seismic lines (*ω*_i_ = 0.359), followed by canopy cover and distance to forest edges and streams (*ω*_i_ = 0.266), followed by the global model (*ω*_i_ = 0.149). The averaged model indicated that kill site locations were more likely to be closer to streams when compared to locations of surviving caribou.

**TABLE 2:** Standardized coefficients (*β*) and margins of error for 95% confidence intervals (±95%CI) for models predicting predation risk based on habitat use 30 days, 7 days, and 24 hr prior to caribou being killed, and attributes directly at kill site locations, outside of protected areas in Narraway and Redrock Prairie Creek caribou ranges in east‐central British Columbia and west‐central Alberta between 2008 and 2015.

Caribou data	Habitat use prior to being killed						Kill site locations	
	30 days prior		7 days prior		24 hr prior			
	*β*	±95% CI	*β*	±95% CI	*β*	95% CI	*β*	±95% CI
Distance to road	**0.187** ^a^	**0.029**	0.058^a^	0.071	−0.098^d^	0.129	−0.390^e^	0.635
Distance to pipeline	**−0.450** ^b^	**0.029**	**−0.166** ^c^	**0.055**				
Distance to seismic	**−0.362** ^a^	**0.049**	**−0.268** ^b^	**0.096**	**−0.785** ^b^	**0.257**	−0.900^c^	1.019
Distance to forest edge	−0.024^d^	0.029			**−0.917** ^a^	**0.296**	−0.313^d^	1.121
Distance to stream	**−0.114** ^d^	**0.022**	**−0.269** ^e^	**0.057**	**−0.679** ^c^	**0.190**	**−1.270** ^d^	**1.137**
Canopy cover	−0.028	0.033	−0.021	0.061	−0.020	0.178	0.003	0.874
South aspect	**−0.101**	**0.067**	**−0.232**	**0.141**	**−1.023**	**0.523**		
Slope	−0.003	0.004	0.065	0.067	0.142	0.184		
TPI							−1.705	1.868
C‐statistic (SE)	0.682 (0.015)		0.641 (0.030)		0.866 (0.080)		0.730 (0.309)	
*r*_s_ (range)	0.708 (0.612–0.749)		0.183 (0.097–0.241)		0.648 (0.531–0.680)		0.490 (0.454–0.596)	

Note

Models were built by comparing GPS locations from nonsurviving caribou to those of caribou from the same herd range that survived during the same time period. Variables are described in Table 1. The most informative spatial scales for all distance‐to‐feature variables are indicated by footnotes. Coefficients with 95% CIs (*β* ± margin of error for 95%CI) that do not overlap zero are indicated in bold. Coefficients and margins of error for kill site locations were model averaged. C‐statistics (with associated SE) and mean and ranges of *r*_s_ values from k‐fold cross‐validation are also shown.

^a^ Distance in m.

^b^ Decay distance, constant at 10 km.

^c^ Decay distance, constant at 5 km.

^d^ Decay distance, constant at 1 km.

^e^ Decay distance, constant at 2 km.

Coefficients for models within protected areas are in Table 3. Within protected areas, the most parsimonious model (*ω*_i_ = 1.00) predicting predation risk during the 30 days prior to caribou being killed indicated that nonsurviving caribou were more likely to be closer to alpine habitat and streams, farther from forest edges, and less likely to be on south‐facing slopes when compared to surviving caribou. The most parsimonious model (*ω*_i_ = 1.00) predicting predation risk during the 7 days prior to caribou being killed also indicated that nonsurviving caribou were more likely to be closer to alpine habitat, farther from forest edges, and less likely to be on south‐facing slopes when compared to surviving caribou. The most parsimonious model (*ω*_i_ = 1.00) predicting predation risk during the 24 hr prior to caribou being killed indicated that nonsurviving caribou were more likely to be closer to alpine habitat, but farther from streams when compared to surviving caribou. The most parsimonious model predicting predation risk at kill site locations included distance to streams, canopy cover, and TPI (*ω*_i_ = 0.459), followed by streams and canopy cover (*ω*_i_ = 0.289), and TPI (*ω*_i_ = 0.252); however, the averaged model revealed no differences between distance to streams, canopy cover, or TPI values at kill site locations when compared to locations of surviving caribou.

**TABLE 3:** Standardized coefficients (*β*) and margins of error for 95% confidence intervals (±95%CI) for models predicting predation risk based on habitat use 30 days, 7 days, and 24 hr prior to caribou being killed and attributes directly at kill site locations, within protected areas in Narraway and Redrock Prairie Creek caribou ranges in east‐central British Columbia and west‐central Alberta between 2008 and 2015.

Caribou data	Habitat use prior to being killed						Kill site locations	
	30 days prior		7 days prior		24 hr prior			
	*β*	95% CI	*β*	95% CI	*β*	95% CI	*β*	95% CI
Distance to alpine	**−0.933** ^a^	**0.069**	**−0.913** ^b^	**0.145**	**−0.960** ^a^	**0.416**		
Distance to forest edge	**0.469** ^a^	**0.057**	**0.143** ^a^	**0.108**				
Distance to stream	**−0.154** ^c^	**0.061**			**1.211** ^c^	**0.372**	−0.478^b^	1.368
Canopy cover							−1.789	3.060
South aspect	**−0.299**	**0.131**						
Slope			**−0.383**	**0.110**				
TPI							−1.912	2.619
C statistic (SE)	0.763 (0.024)		0.782 (0.047)		0.890 (0.118)		0.769 (0.329)	
*r*_s_ (range)	0.958 (0.855–1.000)		0.871 (0.810–0.958)		0.663 (0.572–0.717)		0.357 (0.309–0.482)	

Note

Models were built by comparing GPS locations from nonsurviving caribou to those of caribou from the same herd range that survived during the same time period. Variables are described in Table 1. Coefficients with 95% CIs (*β* ± margin of error for 95% CI) that do not overlap zero are indicated in bold. The most informative spatial scales for all distance‐to‐feature variables are indicated by footnotes. Coefficients and margins of error for kill site locations were model averaged. C‐statistics (with associated SE) and mean and ranges of *r*_s_ values from k‐fold cross‐validation are also shown.

^a^ Decay distance, constant at 1 km.

^b^ Decay distance, constant at 2 km.

^c^ Distance in m.

## 4 DISCUSSION

Unsustainable caribou predation resulting from habitat disturbance is the most significant and immediate threat to central mountain caribou populations (Environment Canada, 2014; Environment and Climate Change Canada, 2018). Therefore, there is an urgent need to restore habitat, and much recent work has focused on seismic line restoration (Finnegan et al., 2018; van Rensen et al., 2015; Tattersall et al., 2019). However, although the link between anthropogenic disturbance and caribou population declines has been well studied, limited research has assessed the influence of specific types of linear disturbance features on caribou predation risk. By assessing caribou predation risk at multiple spatio‐temporal scales and in relation to different types of linear features, we found that predation risk did differ among linear feature types. Specifically, we found that pipelines contributed to predation risk at larger spatio‐temporal scales, seismic lines contributed to predation risk at all scales of habitat use prior to caribou being killed, and natural linear features (i.e., streams) were linked to predation risk both prior to caribou being killed and at kill site locations. Within protected areas, alpine habitat and terrain were important predictors of predation risk, but proximity to alpine habitat increased predation risk rather than serving as a refugia. Our results provide further information regarding the impacts of linear disturbance features on caribou and provide novel insights into the relative impacts of different linear features on caribou predation.

Outside of protected areas, we found that predation risk increased closer to seismic lines across all three spatio‐temporal scales of habitat use. Previous research has shown that seismic lines are used by bears and wolves (Dickie et al., 2019; Finnegan, Pigeon, et al., 2018; Tigner et al., 2014), increase predator travel speeds and encounter rates with prey (Dickie, Serrouya, McNay, et al., 2017; McKenzie et al., 2012; Mumma et al., 2017), and are a source of forage for primary prey species (e.g., moose) and bears (Finnegan, MacNearney, et al., 2018). By assessing habitat use prior to predation‐caused caribou mortalities, our results provide direct evidence that predation risk for caribou is higher near seismic lines. Based on the established link between anthropogenic disturbance and increased caribou predation rates, seismic lines have long been identified as a restoration priority for caribou ranges in Alberta and British Columbia (Environment Canada, 2014; Government of Alberta, 2017a). Our results provide further evidence that the restoration of seismic lines should be an immediate priority to contribute toward caribou recovery in central mountain caribou ranges.

During caribou habitat use 30 days and 7 days prior to being killed, we also found that predation risk increased closer to pipelines. To our knowledge, ours is the first study to assess the relationship between pipelines and habitat use prior to predation‐caused caribou mortalities. Previous research assessing caribou predation risk took place in areas where pipelines were not present (Leblond et al., 2013), grouped all linear disturbance features together (DeMars & Boutin, 2018; James & Stuart‐Smith, 2000), did not include pipelines in their assessment (Mumma et al., 2017), or did not assess habitat use prior to caribou being killed (Latham, Latham, Boyce, et al., 2011). As with seismic lines, bears and wolves use pipelines (Dickie et al., 2019; Finnegan, MacNearney, et al., 2018; McKay et al., 2014), pipelines contain forage for primary prey species and bears (MacDonald et al., 2020), and the presence of pipelines likely increases the spatial overlap and probability of encounter between predators and caribou (Frey et al., 2017; Whittington et al., 2011). Pipelines may also facilitate caribou predation by increasing predator search rates, as reported for roads and seismic lines (Dickie, Serrouya, McNay, et al., 2017; Latham, Latham, Boyce, et al., 2011; McKenzie et al., 2012). In addition, vegetation is cleared on active pipelines in Alberta to facilitate pipeline maintenance and pipeline visibility from the air (Alberta Energy Regulator, 2016). Use of linear disturbance features by predators has been linked to ease of travel, with wolves and bears selecting linear disturbance features with shorter vegetation (Dickie et al., 2017; Finnegan, Pigeon, et al., 2018; Tigner et al., 2014). Therefore, the combined effect of consistently short vegetation and low levels of human use on pipelines likely makes pipelines attractive travel corridors for predators, increasing predation risk for caribou in areas near pipelines.

While predation risk increased closer to pipelines and seismic lines, we found that during the 30 days prior to their being killed, caribou predation risk decreased closer to roads. These results conflict with previous research reporting that predators such as wolves and grizzly bears select for roads (e.g., Dickie et al., 2019; Roever et al., 2008; Whittington et al., 2011) and that the probability of caribou encountering wolves increases in areas with higher road densities (Leblond et al., 2013; Mumma et al., 2017). Our results did not suggest a functional response of predation risk to road density, as the interaction between distance to road and road density was not selected in our final models, but the relationship we observed between proximity to roads and predation risk could be related to human activity levels on roads. Although bears and wolves use roads (Dickie, Serrouya, McNay, et al., 2017; Roever et al., 2008; Whittington et al., 2011), research also indicates that bears, wolves, and cougars avoid linear disturbance features with high levels of human activity (Dickie, Serrouya, McNay, et al., 2017; Muhly et al., 2011; Northrup et al., 2012). Leblond et al. (2013) also found that in areas of high total road density, the probability of caribou dying from predation depended on human activity levels on the roads (i.e., active versus unmaintained roads) rather than simply being related to the presence of roads. When high levels of human activity displace predators, the result can be localized decreased predation risk (Berger, 2007; Hebblewhite & Merrill, 2009; Muhly et al., 2011). Road activity data were not available at the time of our analysis, but future analyses with additional data could provide insight into the links between human activity levels on roads and caribou predation risk.

We also found that predation risk outside of protected areas was higher for caribou using habitat closer to streams, and kill sites were also closer to streams than surviving caribou locations. This result is consistent with previous research indicating that grizzly bears, cougars, and wolves select streams and riparian areas for foraging, cover, and movement (DeCesare, 2012a; Dickson et al., 2005; Phoebus et al., 2017), and that prey vulnerability to being killed is higher near streams (Kunkel & Pletscher, 2000; Nielson & Boutin, 2017). Within protected areas, caribou predation risk was linked to natural landscape characteristics previously associated with predators (e.g., streams, lower angle slopes). However, in contrast to previous work reporting that caribou predation risk from wolves decreases at higher elevations (Apps et al., 2013; Mumma et al., 2017; Whittington et al., 2011), we found that predation risk increased closer to alpine habitat. It is possible that in a multi‐predator system like our study area, alpine habitat may not be an effective refuge for caribou. Cougars may use higher elevations to avoid overlap with wolves (Bartnick & Van Deelen, 2013; Kortello et al., 2007), and within our study area, grizzly bears use alpine habitat during summer (Nielsen et al., 2006). When prey species spatially avoid the risk of one predator, they may become more vulnerable to other predator species; caribou using high elevation habitat to avoid wolves could be at higher risk of encounters with bears or cougars (Atwood et al., 2009; Leblond et al., 2016; Pinard et al., 2012).

As in previously published research, we found no direct link between kill site locations and linear disturbance features (e.g., Latham, Latham, Boyce, et al., 2011; Mumma et al., 2017). However, our models of caribou habitat use prior to being killed indicate that predation risk increased closer to pipelines and seismic lines. We also found that landscape features such as pipelines had more influence on caribou predation risk at larger spatio‐temporal scales, while landscape features such as seismic lines, streams, and forest edges had more influence on predation risk just before caribou were killed (<24 hr) and at kill site locations. Our results highlight the importance of considering multiple spatio‐temporal scales (i.e., both kill site locations and premortality habitat use) when investigating caribou predation risk. In addition, the divergent influences of pipelines and seismic lines versus roads revealed by our analysis highlight the importance of assessing the separate influences of specific linear disturbance features in predation risk analyses.

We recognize that our study had limitations. Model validation indicated low accuracy and poor predictability for models at some spatio‐temporal scales, and models at kill sites included uninformative variables. Poor model performance is likely due to the small number of predation events in the datasets (outside of protected areas, *n* = 12; within protected areas, *n* = 6). We were also unable to assess the influence of additional factors known to influence predation risk (e.g., caribou health and age, winter snow conditions, human activity levels, caribou population size, local population sizes of predators, and alternate prey), as these data were unavailable for our study area across the years of our study (2008–2015). Inclusion of these variables in future analyses could improve predictability of predation risk models and provide further insight into the mechanisms affecting caribou survival. In addition, although the majority (83%) of mortalities in our study took place prior to wolf control activities in the Narraway and Redrock Prairie Creek caribou ranges, it is possible that our results were affected by changes in wolf abundance and distribution resulting from wolf control both within our study area and in adjacent ranges. Predator species within our study area (i.e., bears, wolves, and cougars) demonstrate diverse habitat selection patterns (DeCesare, 2012a; Knopff, 2011; Nielsen et al., 2006), and variables predicting caribou predation risk can depend on the specific predators involved (Apps et al., 2013). Separating out the different predator species in our analysis could eliminate the potential influence of changes in wolf populations and reveal stronger associations between landscape features and caribou predation risk from specific predators. Unfortunately, for this study, we were unable to partition mortality data by predator species due to the small sample size and the uncertainty in determining the predator species responsible for the initial kill. Regardless of the limitations of our current dataset, we believe that the predation risk patterns we observed in this study apply to overall caribou predation risk in the Narraway and Redrock Prairie Creek caribou populations at the time of our study, and our findings have important implications that may be applied in ongoing recovery efforts for this declining species. Particularly considering the precipitous declines in many caribou populations (Hervieux et al., 2013, Environment Canada, 2014, Government of Alberta, 2017a), we believe it is important to report these results at the current time, rather than delaying until a larger dataset can be obtained.

## 5 CONCLUSIONS

Landscape managers require specific information to be most efficient and effective at habitat restoration for caribou conservation. A large body of research has focused on the indirect mechanisms of increased caribou predation risk associated with anthropogenic disturbances (e.g., DeCesare, 2012b; Dickie, Serrouya, McNay, et al., 2017; Whittington et al., 2011), but few studies have provided evidence of direct relationships between disturbance features and caribou predation. By investigating whether specific linear disturbance features were directly associated with caribou predation events, we showed that pipelines and seismic lines increased caribou predation risk. Focusing restoration and habitat management to reduce predator use of pipelines and seismic lines, including mitigations that reduce predator travel (e.g., Keim et al., 2019), could help decrease caribou predation risk. However, our results also indicate that the relationship between linear disturbance features and predation risk is complex, and may differ across specific linear disturbance features. Finally, by considering both habitat use prior to caribou being killed and characteristics at kill sites, our study revealed relationships between predation risk and habitat, terrain, and linear disturbance features that would not have been evident by considering the kill site location alone.

## CONFLICT OF INTEREST

None declared.

## AUTHOR CONTRIBUTIONS

**Tracy McKay:** Conceptualization (equal); data curation (supporting); formal analysis (lead); funding acquisition (supporting); investigation (equal); methodology (equal); validation (lead); writing–original draft (lead); writing–review and editing (lead). **Karine Pigeon:** Investigation (equal); methodology (equal); writing–original draft (equal); writing–review and editing (equal). **Terry Larsen:** Conceptualization (equal); data curation (supporting); writing–review and editing (supporting). **Laura Finnegan:** Conceptualization (lead); data curation (lead); funding acquisition (lead); investigation (equal); methodology (equal); project administration (lead); writing–original draft (supporting); writing–review and editing (supporting).

## Data Availability

Woodland caribou are listed as a threatened species under federal legislation, and locations collected with GPS telemetry are considered confidential. Given that caribou display high fidelity to home ranges and seasonal habitats, they are considered particularly vulnerable to hunting and anthropogenic disturbance. Making telemetry locations publicly available could pose a serious risk to federal and provincial caribou recovery efforts, and it is under this discretion that we do not provide the GPS telemetry data used in this manuscript.

## APPENDIX 1

### Candidate variables and models by category

Candidate variables and models in each category used to predict caribou predation risk (based on habitat use 30 days, 7 days, and 24 hr prior to mortality events and at kill site locations) outside of (Table A1) and within (Table A2) protected areas in Redrock Prairie Creek and Narraway caribou ranges in east‐central British Columbia and west‐central Alberta during 2008–2015. Variables include D_edge (distance to nearest forest edge), dStrm3 (distance to nearest stream, Strahler order ≥ 3), dStrmAll (distance to nearest stream, any size), dAlp (distance to nearest alpine habitat), cc (canopy cover), lc (landcover), (forest = presence/absence of forest), DRd (distance to nearest road), dPipe (distance to nearest pipeline), dSeismic (distance to nearest seismic line), and dPS (distance to nearest pipeline or seismic line). All distance‐to‐feature variables were calculated at five spatial scales, including Euclidian distance to features (m) and four exponential decay distances to represent the diminishing effect of features on animal response at greater distances, where the effect of distance becomes approximately constant at 1 km, 2 km, 5 km, and 10 km, represented in the final model variables as .m, 0.1, 0.2, 0.5, and 0.10, respectively.

**TABLE A1:** Candidate variables and models used to predict caribou predation risk outside of protected areas

Model category	Candidate variables	Spatio‐temporal scale	Most parsimonious model in category
Habitat	D_edge, dStrm3, dStrmAll, dAlp, cc, lc, forest	30 days prior 7 days prior 24 hr prior Kill site location	D_edge.1 + dStrm3.1 + cc dStrm3.2 + cc D_edge.m + dStrmAll.5 + cc D_edge.1 + dStrmAll.1 + cc1
Terrain	Aspect, CTI, DEM, Slope, TPI	30 days prior 7 days prior 24 hr prior Kill site location	Aspect + DEM +Slope Aspect + DEM +Slope Aspect + DEM +Slope TPI
Linear disturbance features	DRd, dPipe, dSeismic, dPS	30 days prior 7 days prior 24 hr prior Kill site location	DRd.m + dPipe.10 + dSeismic.m DRd.m + DpipeNR.5 + dSeismic.10 DRd.1 + dSeismic.10 DRd.2 + dSeismic.5

**TABLE A2:** Candidate variables and models used to predict caribou predation risk within protected areas

Model category	Candidate variables	Spatio‐temporal scale	Most parsimonious model in category
Habitat	D_edge, dStrm3, dStrmAll, dAlp, cc, lc, for	30 days prior 7 days prior 24 hr prior Kill site location	D_edge.1 + dStrmAll.m + dAlp.1 D_edge.1 + dAlp.2 dStrm3.m + dAlp.1 dStrm3.2 + cc
Terrain	Aspect, CTI, DEM, Slope, TPI	30 days prior 7 days prior 24 hr prior Kill site location	Aspect + DEM DEM + Slope DEM TPI
